# Chemotherapy in geriatric patients with poor performance status small cell lung cancer: Series from a tertiary care center

**DOI:** 10.1002/agm2.12205

**Published:** 2022-04-16

**Authors:** Deepak Sundriyal, Parmod Kumar, Ujjawal Kumar, Amit Sehrawat

**Affiliations:** ^1^ Department of Medical Oncology, Hematology All India Institute of Medical Sciences Rishikesh India; ^2^ Department of Internal Medicine All India Institute of Medical Sciences Rishikesh India

**Keywords:** advanced‐stage cancer, geriatric patients, poor performance status, small cell lung cancer

## Abstract

Geriatric age group patients with poor performance status and advanced stage cancer are often denied chemotherapy. In this series of cases, we demonstrated that systemic anti‐cancer therapy can be considered in these patients after a meticulous modification of the chemo‐protocol.

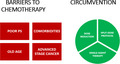

## 1 INTRODUCTION

Although no age is a bar from the development of cancer, approximately half of all the malignancies and more than two‐thirds of all the malignancy‐related deaths are encountered in the geriatric population. These numbers are expected to increase as the world population is aging. Similar trends are expected for India, and that will create a big challenge to the health care delivery system.^1^, ^2^ Geriatric age is considered a barrier for the recruitment of patients in the cancer treatment–related trials due to the fear of poor tolerance and efficacy. As the general principles and the modalities of cancer treatment are essentially the same in the younger and geriatric population, the latter requires a meticulous assessment for the optimum therapeutic goal of improved quality of life, palliation of symptoms, and prolonging survival. Cancer chemotherapy in geriatric patients with advanced‐stage malignancy and poor functional reserve poses a specific challenge. We present our series of five patients of advanced‐stage small cell lung cancer (SCLC), who presented to us with a poor performance status (PS) and were treated with a modified chemotherapy protocol.

## 2 PATIENTS AND METHODS

We retrospectively reviewed our data of patients with SCLC who presented to us between April 2019 and September 2020. Geriatric patients with advanced‐stage SCLC, who were otherwise not a candidate for systemic anticancer treatment due to poor PS and comorbidities, were included in the study. Data were retrieved from computerized records and the medical records department. Clinico‐pathological and geriatric assessment parameters including the stage, Eastern Cooperative Oncology Group‐PS (ECOG‐PS), comorbidities, smoking pattern, Activities of Daily Living (ADL), Instrumental Activities of Daily Living (AIDL), polypharmacy, history of falls, family support, and chemo‐toxicity score were noted. A total of five patients received the modified weekly protocol of etoposide and carboplatin after a detailed discussion with the family members. Intravenous etoposide and carboplatin were administered weekly in the dose of 80–100 mg/m^2^ and AUC‐2, respectively. Prophylactic growth factor support was given to all patients. Additional protocol modifications were in the form of 15%–20% dose reduction after chemotherapy‐related toxicities and the use of single‐agent platinum in first cycle. Responses, survival outcomes, improvement in PS, and the cause of death were noted. Toxicities were studied as per the Common Terminology Criteria for Adverse Events (CTCAE v5.0). Permission for the retrieval of records and approval for the study was taken as per the institutional protocol.

## 3 RESULTS

The mean age of the group was 65.2 (60–69) years. Three patients were females. All of them presented to us with stage 4 disease and with an ECOG‐PS of 4. Two patients had chronic obstructive pulmonary disease while one of them suffered from deep venous thrombosis and tumor‐related superior vena cava syndrome at the time of presentation. ADL assessment by the Katz tool was suggestive of high dependence on a caregiver.^3^ Lawton and Brody’s scale assessment of IADL was also suggestive of higher dependence on a caregiver.^4^ None of the patients had a history suggestive of polypharmacy or falls. Four patients had a medium risk score(50%–54%) of 6,6,8, and 9, while one patient had a high risk (77%) score of 11 on the Cancer and Aging Research Group (CARG) chemo‐toxicity tool.^5^

The mean number of cycles received by each patient was 5.6 (range 4–8). Clinical response in the form of improvement in pain and dyspnea was seen within 7–10 days in all patients. Four patients demonstrated partial response (PR) after three cycles while one had stable disease (SD). Median progression‐free survival (PFS) was 137 days while median overall survival (OS) was 164 days. All of them demonstrated an improvement in PS. The cause of death was progressive disease in two patients and massive pulmonary embolism in one patient. One patient each was continuing on the same protocol and second line chemotherapy (irinotecan based; Table 1).

**TABLE 1:** Clinico‐pathological features and treatment outcome

Clinico‐Pathological features and treatment outcome	Patient 1	Patient 2	Patient 3	Patient 4	Patient 5
Age (years)	65	60	69	65	67
Sex	F	F	M	M	F
Stage of the disease	4	4	4	4	4
ECOG‐PS	4	4	4	4	4
Comorbidities	None	None	COPD, DVT, SVC syndrome	None	COPD
Smoking	Passive	Active	Active	Active	Active
ADL	2/6	2/6	2/6	1/6	2/6
IADL	1/8	1/8	2/5	1/5	1/8
Toxicity Score	9 (medium)	8 (medium)	6 (medium)	6 (medium)	11 (high)
Polypharmacy	No	No	No	No	No
Falls	None	None	None	None	None
Family support	Yes	Yes	Yes	Yes	Yes
Additional protocol modification	Dose reduction	None	Single agent platinum in the first cycle	None	None
No. of cycles received	5	6	8	5	4
Response	PR	PR	PR	PR	SD
PFS (days)	137	103	NR	317	97
OS (days)	177	164	NR	NR	103
PS after chemotherapy	2	2	2	1	3
Toxicity (Grade ¾)	Anemia, Neutropenia	Anemia, Thrombocytopenia	Thrombocytopenia, Neutropenia	Thrombocytopenia	Neutropenia, Oral mucositis, Fatigue
Cause of death	PD	PD	NA	NA	PE

Abbreviations: ADL, Activities of daily living; COPD, chronic obstructive pulmonary disease; DVT, deep venous thrombosis; ECOG‐PS, Eastern cooperative oncology group‐performance status; IADL, instrumental activities of daily living; NA, not applicable; NR, not reached; OS, overall survival; PE, pulmonary embolism; PFS, progression free survival; PR, partial resonse; PS, performance status; SD, stable disease; SVC, superior vena cava.

Grade 3–4 toxicities included anemia in two patients, neutropenia, and thrombocytopenia in three patients each, while oral mucositis and fatigue in addition to neutropenia were observed in the patient with a high‐risk CARG toxicity score leading to dose reduction. A delay ranging from 1–4 days was observed in the initiation of subsequent chemotherapy. Overall, chemotherapy with the modified protocol and further modifications was well tolerated by all patients.

## 4 DISCUSSION

Our results provide some evidence that a weekly modified protocol of etoposide/platinum can be effective and safe in geriatric patients with advanced‐stage SCLC who are otherwise unfit after a geriatric assessment due to poor PS, comorbidities, or anticipated chemotherapy‐related toxicities. The conventional regimen of a 3‐weekly etoposide/platinum combination has been studied in patients of SCLC with poor performance status. Aida Y. et al treated 12 patients (elderly as well as young) of SCLC of ECOG‐PS of 3 and 4 with the conventional 3‐weekly regimen. They demonstrated an improvement in PS in seven patients with a PR elicited in five patients. Median OS was 147 days.^6^ Another study of the outcome of treatment in patients with SCLC in poor PS of 3 and 4 demonstrated a median OS of 5.1 months. However, the median OS was poor (2.7 months) for the patients (*n* = 6) with a PS of 4. Carboplatin (AUC5, Calvert formula) was administrated intravenously on day one, and etoposide (240 mg/m^2^) was given orally daily from day one to three.^7^ A previous study with weekly etoposide and platinum doublet in patients of SCLC unfit for standard regimen found the weekly regimen as a valid option with no excess toxicity. The clinical benefit rate was achieved by 57% of the patients in their study.^8^

Geriatric patients who have a poor PS of 3 and 4 are often excluded in the clinical trials because of the fear of treatment‐related toxicity and considered for best supportive care. Furthermore, the usual pattern of standard care is often jeopardized by comorbidities, frailty, and the lack of social support.

However, efforts must be put to identify the subset of elderly patients who can be a candidate for systemic chemotherapy and how the chemotherapy protocol can be modified to provide the necessary palliation and prolongation of survival simultaneously improving or preserving the quality of life.

Geriatric patients who have been diagnosed with highly chemo‐sensitive solid organ tumors like SCLC, ovarian cancer, or neuroendocrine tumors may be considered for a trial of chemotherapy. A meticulous geriatric assessment including but not limited to the functional status, comorbidities, polypharmacy, frailty, family support, and chemo‐toxicity score can further identify the patients who can be expected to tolerate the chemotherapy. Furthermore, the chemo‐protocol can be modified in several ways including splitting the multiple‐day protocol into a weekly protocol, reducing the dose of chemotherapeutic agents, and beginning with a single agent in the first few sittings of chemotherapy. Growth factor support can be added. A clinical response can be expected as early as the seventh day in highly chemo‐sensitive tumors and this can serve as a benchmark to further continue or discontinue the therapy. Moreover, toxicities are expected to be mild as compared to a standard conventional protocol and can be easily managed.

## 5 CONCLUSIONS

1. Geriatric patients with poor PS and diagnosed with highly chemo‐sensitive tumors may be considered for systemic anti‐cancer therapy.
2. A meticulous geriatric assessment for the selection and the modification in the conventional chemo‐protocol are warranted. Prospective clinical trials in the geriatric population with poor PS are the way forward.

## ACKNOWLEDGMENTS

We acknowledge the contribution from the geriatric oncology clinic.

## CONFLICT OF INTEREST

There are no conflict of interest to disclose.

## AUTHOR CONTRIBUTIONS

Conceptualization: D.S. and P.K. Data collection: D.S., A.S., and U.K. Data analysis: D.S., U.K., and P.K. Drafting the article: D.S., A.S., and U.K. Critical appraisal and final approval: All authors.

## PATIENT CONSENT STATEMENT

Formal consent was not required due to the retrospective nature of the study.
